# The STAT3/TIMP1 inhibitor silibinin overcomes secondary immunoresistance to pembrolizumab in brain metastases from METex14 skipping mutated non-small cell lung cancer: a case report

**DOI:** 10.3389/fmed.2025.1612327

**Published:** 2025-07-09

**Authors:** Joaquim Bosch-Barrera, Elia Sais, Eduard Teixidor-Vilà, Carmen Vásquez-Dongo, Alejandro Hernandez-Martínez, Alvaro Romera, Mel.lina Pinsach-Abuin, Bernat del Olmo, Glòria Oliveras, Emma Polonio-Alcalá, Sara Verdura, Miriam Soriano-Gamero, Victor Pineda, Hugo Rosales, Javier A. Menendez

**Affiliations:** ^1^Medical Oncology Department, Catalan Institute of Oncology, Dr. Josep Trueta University Hospital, Girona, Catalonia, Spain; ^2^OncoGIR-PRO (Precision Oncology Group), Girona Biomedical Research Institute (IDIBGI-CERCA), Girona, Catalonia, Spain; ^3^Medical Science Department, School of Medicine, University of Girona, Girona, Catalonia, Spain; ^4^Department of Pathology, Dr. Josep Trueta University Hospital, Girona, Catalonia, Spain; ^5^Molecular Diagnostics and Precision Medicine Unit, Girona Territorial Clinical Laboratory, Dr. Josep Trueta University Hospital, Girona, Catalonia, Spain; ^6^Program Against Cancer Therapeutic Resistance (ProCURE), Catalan Institute of Oncology, Girona, Catalonia, Spain; ^7^Metabolism & Cancer Group, Girona Biomedical Research Institute (IDIBGI), Girona, Catalonia, Spain; ^8^Department of Radiology, Diagnostic Imaging Institute, Dr. Josep Trueta University Hospital, Girona, Catalonia, Spain; ^9^Department of Radiotherapy, Catalan Institute of Oncology, Dr. Josep Trueta University Hospital, Girona, Catalonia, Spain

**Keywords:** silymarin, immunotherapy, natural product, lung adenocarcinoma, brain radiotherapy

## Abstract

**Background:**

Approximately 20% of patients with non-small cell lung cancer (NSCLC) are diagnosed with brain metastases (BM), which are associated with poor prognosis. Pembrolizumab has shown promising results in advanced NSCLC with PD-L1 ≥ 50%, including patients with BM. Silibinin is a flavonolignan with known blood-brain barrier permeability and anti-BM activity associated with inhibition of the STAT3/TIMP1 signaling axis. To the best of our knowledge, this is the first clinical evidence of combining silibinin with pembrolizumab to achieve a durable partial response in BM, lasting over 9 months.

**Case Report:**

We present the case of a 72-year-old male with stage IVB lung adenocarcinoma and BM, who achieved durable intracranial tumor control with a combination of pembrolizumab and silibinin supplementation. Initial treatment with brain hypofractionated stereotactic radiotherapy and pembrolizumab led to a 14-month partial response. Progression occurred with a new jejunal metastasis and increasing temporal brain lesion. After declining whole-brain radiotherapy, the patient continued pembrolizumab with silibinin (630 mg/day), on a compassionate basis. At 2 months, a partial response in the temporal lobe lesion was observed, and at 9 months, nearly complete intracranial response was achieved with no extracranial progression. Molecular analysis revealed high PD-L1 expression and a METex14, potentially enhancing the response to immunotherapy.

**Conclusion:**

This case highlights the potential of silibinin as an adjuvant therapy to enhance anti-PD-1 efficacy in brain metastases, possibly by targeting STAT3/TIMP1-driven immunosuppressive astrocytes. Further investigation of the role of silibinin in improving immunotherapy outcomes in advanced lung cancer patients with BM is required.

## 1 Introduction

Approximately 10%–25% of patients with non-small cell lung cancer (NSCLC) are diagnosed with brain metastases (BMs), which significantly contribute to morbidity and mortality and are associated with reduced performance status and quality of life (1). Despite limited treatment options, patients with BMs may benefit from systemic immunotherapy.

A pooled analysis of the KEYNOTE-001, -010, -024, and -042 trials demonstrated a clear survival advantage of pembrolizumab over chemotherapy in patients with PD-L1–positive NSCLC and BMs. The greatest benefit was observed in patients with a PD-L1 tumor proportion score (TPS) ≥ 50%, showing a median overall survival (OS) of 19.7 months [95% confidence intervals (CI): 12.1–31.4] compared to 9.7 months with chemotherapy (95% CI: 7.2–19.4; HR: 0.67, 95% CI: 0.44–1.02) (2). However, the heterogeneous genomic and immunophenotypic landscape of NSCLC may influence the efficacy of immune checkpoint inhibitors (ICIs) in the context of BMs (3).

Silibinin, a flavonolignan derived from the seeds of *Silybum marianum* (milk thistle), is a major component of the crude extract silymarin and has demonstrated the ability to cross the blood-brain barrier (BBB) (4–6). Preclinical and early clinical studies have exhibited that silibinin is highly active against BMs in *ex vivo*, animal models, and NSCLC patients (5, 6). Its mechanism of action is primarily attributed to the inhibition of the STAT3/TIMP1 signaling pathway, which is hyperactivated in a subset of the phospho-STAT3+ reactive astrocytes (RA) surrounding BMs (7, 8). By inhibiting STAT3 activation (7–9), silibinin disrupts the immunosuppressive functions of RAs that prevents both adaptive (CD8+ T cells) and innate (macrophages/microglia) immune responses in BMs (9). Furthermore, silibinin suppresses the secretion of TIMP1 from these RA, thereby modulating the function of CD63+ CD8+ T cells (10). The combination of ICIs with silibinin has demonstrated enhanced antitumor efficacy in both murine and human BM models (10).

Here, we report a unique case of 72-year-old man with PD-L1-high NSCLC and BMs who achieved a durable partial intracranial response, lasting over 9 months, through the combination of silibinin and pembrolizumab.

## 2 Case description

**FIGURE 1:**
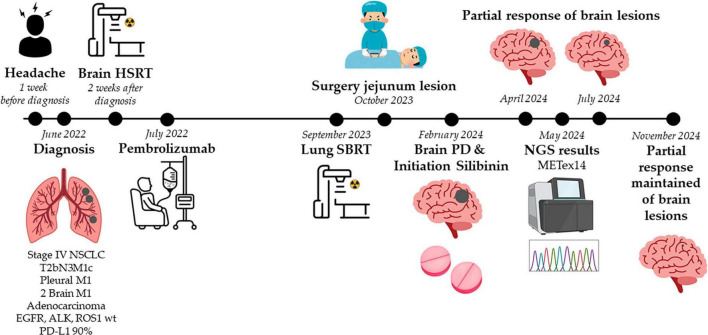
Timeline chart detailing the case. HSRT, hypofractionated stereotactic radiotherapy; METex14, *MET* exon 14 skipping mutation; NGS, next-generation sequencing; NSCLC, non-small cell lung cancer; PD, progression disease; SBRT, stereotactic body radiation therapy.

A detailed timeline of the case is shown in Figure 1.

**FIGURE 2:**
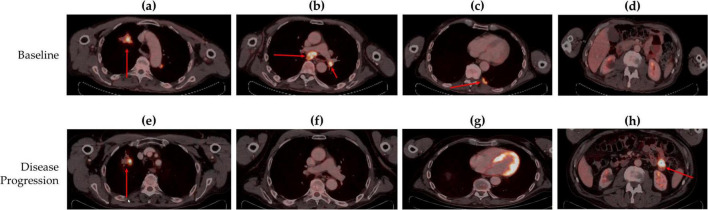
Body Fluorine-18 fluorodeoxyglucose positron emission tomography–computed tomography (18-FDG PET-CT) scans of **(a)** right upper lung lobe lesion at baseline, **(b)** contralateral hilar (cN3) and subcarinal (cN2) metabolically active lymphadenopathy at baseline, **(c)** right pleural nodal lesion at baseline, **(d)** no lesion in the jejunum previous at disease progression, **(e)** right upper lung lobe lesion at disease progression, **(f)** contralateral hilar (cN3) and subcarinal (cN2) lymphadenopathy with complete response at follow-up, **(g)** right pleural nodal lesion with complete response at follow-up, and **(h)** a new lesion detected in the jejunum at disease progression. Red arrows indicate the lesion.

A 72-year-old Caucasian man, a former smoker, visited our emergency department in June 2022 complaining of headache. Brain computed tomography (CT) revealed two masses, subsequently confirmed by magnetic resonance imaging (MRI): one in the right parietal lobe (16 mm) and the other in the right temporal lobe (17 mm). Following this, we performed a whole-body positron emission tomography (PET)-CT scan to assess the potential presence of additional lesions. PET-CT images revealed a 46 mm lesion in the right upper lobe of the lung (RULL) [maximum standardized uptake value (SUVmax) 8.6] (Figure 2a), pathological subcarinal (SUVmax 12.8) and bilateral hilar lymph nodes (SUVmax 10.6) (Figure 2b), and a 26 mm nodule (SUVmax 7.79) in the right pleura (Figure 2c).

A CT-guided biopsy of the lung mass confirmed the diagnosis of adenocarcinoma. The results of Anatomical Pathology showed a PD-L1 TPS of 90%, with negative results for ALK, ROS1, and mutations in *EGFR*, using both tissue and liquid biopsy. Next-generation sequencing (NGS) analysis or EGFR Cobas PCR testing was not possible due to insufficient tumor tissue. Then, the disease was staged as cT2bN3M1c (brain and pleura) – stage IVB adenocarcinoma, with high PD-L1 and no driver mutation detected. No family history of cancer was reported by the patient.

In July 2022, the patient was treated with hypofractionated stereotactic radiotherapy (HSRT) (35Gy in 5 sessions) for the two BM and initiated systemic therapy pembrolizumab (2 mg/kg every 3 weeks). A partial response was achieved at all disease sites and maintained for 14 months.

In September 2023, a follow-up PET-CT scan revealed activity in the RULL mass (SUVmax 16.37 g/ml) (Figure 2e) and complete metabolic response in the other initial lesions (Figures 2f, g). Moreover, a new lesion in the jejunum was observed, suggesting a new tumor (SUVmax 16.97 g/ml) (Figures 2d, h). Brain MRI showed an increase in the size of the right temporal lobe lesion, raising suspicions of radiation necrosis or tumor progression.

As part of the institutional protocol, the case was reviewed by multiple multidisciplinary tumor boards. The Thoracic Tumor Board recommended stereotactic body radiation therapy (SBRT) to the RULL lesion (60Gy in 8 sessions). The Digestive Tumor Board proposed diagnostic abdominal laparoscopy for further evaluation of the jejunal lesion. The Central Nervous System (CNS) Tumor Board also suggested an ^18^F-fluorodopa (FDOPA) PET-CT scan of the brain. The patient underwent all the procedures as recommended by the respective tumor boards.

**FIGURE 3:**
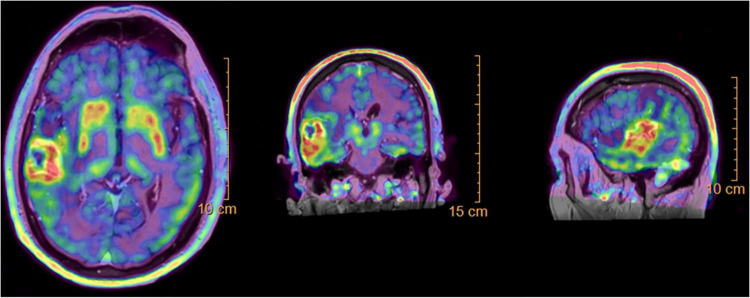
Brain ^18^F-fluorodopa positron emission tomography-computed tomography (FDOPA PET-CT) scan showing hypermetabolic activity in the right temporal lobe lesion, with a maximum standardized uptake value (SUVmax) of 3.38 g/ml, indicating tumor viability and progression.

The jejunal lesion was resected, and histopathological analysis of the surgical specimen confirmed it to be a metastasis from the primary lung adenocarcinoma. Brain FDOPA PET-CT images revealed hypermetabolic activity in the right temporal lobe lesion [SUVmax 3.38 g/ml; Tumor/Striatal Ratio: 1.09 (positive > 1); Tumor/Normal cortex Ratio: 2.11 (positive > 1.3)], findings consistent with tumor viability and progression (Figure 3). Despite these findings, the patient remained asymptomatic, and treatment with pembrolizumab was continued.

**FIGURE 4:**
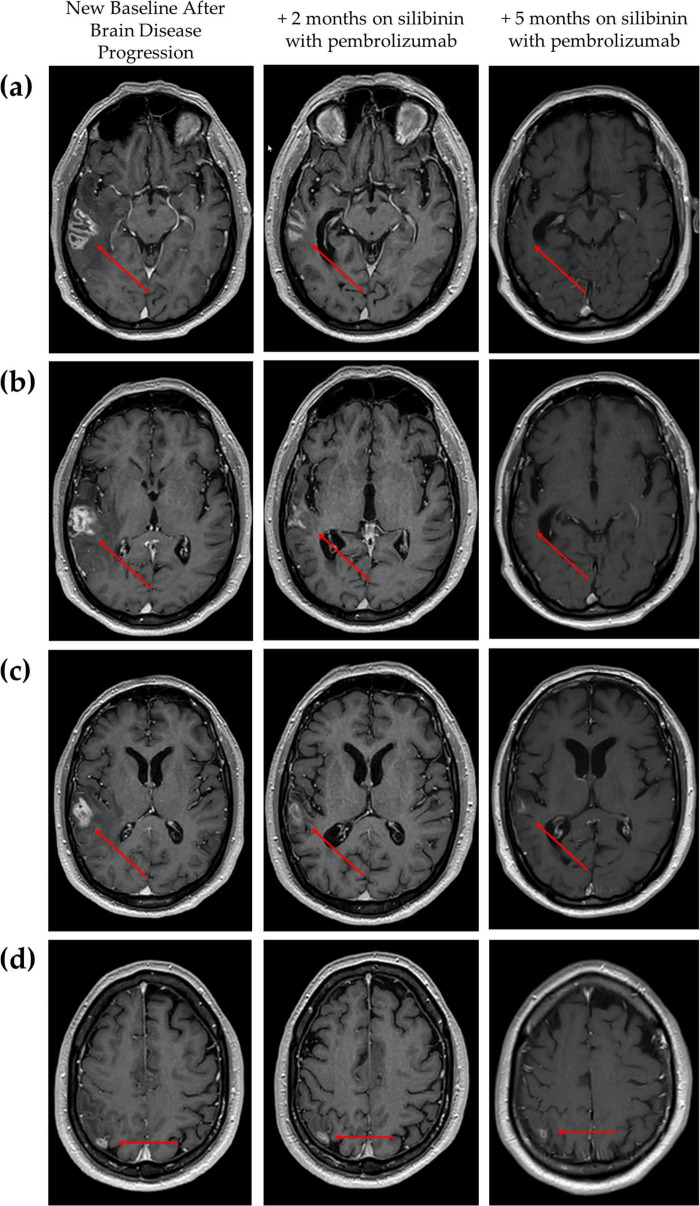
Brain magnetic resonance imaging (MRI) showing the radiological extension of the brain metastases at new baseline (when brain progression was confirmed on pembrolizumab) and evolution after silibinin supplementation with pembrolizumab at 2 and 5 months. **(a–c)** Radiological changes of right temporal lobe lesion over combination treatment using different slices to reflect its heterogeneous nature and **(d)** Radiological changes of right parietal lesion over combination treatment. Red arrows indicate the lesion.

In February 2024, a new follow-up brain MRI (17 months after treatment initiation) showed progression of both brain lesions: the temporal lobe lesion had increased (22 × 29 × 31 mm) (Figures 4a–c, new baseline after brain disease progression) as well as the parietal lobe lesion (11 × 7 mm) (Figure 4d, new baseline after brain disease progression). The CNS Tumor Board ruled out neurosurgical resection due to the aggressive nature of the required surgery and the anticipated poor outcomes. Whole brain radiotherapy (WBRT) with hippocampal sparing was recommended; however, the patient declined WBRT and opted to continue follow-up given the absence of neurological symptoms. At this point, pembrolizumab was continued and we offered the patient compassionate use of silibinin. The patient tolerated the silibinin dose (up to 630 mg/day) without adverse effects.

After 2 months of combination therapy, brain MRI revealed a partial response: the temporal lobe lesion had decreased (20 × 10 × 21 mm) (Figures 4a–c, +2 months on silibinin with pembrolizumab) as well as the parietal lobe lesion (Figure 4d, +2 months on silibinin with pembrolizumab). No evidence of extracranial progression was found on body CT scan. After 5 months of combination treatment, further radiologic improvement was observed, with a reduction in surrounding vasogenic edema and difficulty measuring the temporal lobe lesion due to its irregular morphology (Figures 4a–c, +5 months on silibinin with pembrolizumab). The parietal lobe lesion had also reduced to approximately 7 × 6 mm (Figure 4d, +5 months on silibinin with pembrolizumab). After 9 months of pembrolizumab in combination with silibinin supplementation, the partial brain response was almost complete with no evidence of extracranial relapse. During the combination therapy, no toxicity related to silibinin or pembrolizumab was observed.

In May 2024, additional molecular analysis of the jejunal metastasis revealed persistent high PD-L1 TPS (90%) and two somatic mutations: a *MET* exon 14 skipping mutation [METex14, variant allele frequency (VAF) 49.9%] and a *TP53* p. (Ser241Tyr) mutation (VAF 33.7%). Given the continued clinical benefit from pembrolizumab and silibinin, MET tyrosine kinase inhibitors (e.g., tepotinib or capmatinib) were not initiated, although both are approved in Spain as second-line treatment for metastatic METex14 NSCLC.

The patient provided written informed consent for the publication of this report (Supplementary Figure 1).

## 3 Discussion

Our case highlights the importance of timely integration of NGS analysis to better understand the evolution of BM in the clinical management of NSCLC patients (11). Initially, we were limited by the amount of tumor sampled at diagnosis and the need to start treatment early in a patient with BMs. Subsequent NGS analysis of the surgically resected jejunal metastasis provided additional patient-relevant molecular information (i.e., actionable METex14). However, the results were received when the patient had a confirmed partial response and we decided, together with the patient, to delay any change in therapy until progression was observed. A key limitation of the molecular analysis is that it was conducted on the jejunal metastasis rather than the BMs. Molecular heterogeneity may exist not only between primary tumors and BMs, but also among different metastatic sites within the same patient (12). Importantly, neurosurgical resection of the BMs was not performed, as the CNS Tumor Board considered the procedure too high-risk with a poor risk-benefit profile and consequently, no tissue sample from the BMs was available for analysis. Therefore, while NGS using liquid biopsy could be a valuable option for similar clinical scenarios, access to liquid NGS remains limited in many centers, even though studies suggest it may be a cost-effective approach in our setting (13).

Specifically, molecular analysis of the patient’s jejunal tumor sample revealed a high PD-L1 expression level (90%), the presence of a METex14 mutation (VAF 49.9%), and a *TP53* mutation (VAF 33.7%), as determined by NGS. METex14 is also generally associated with a higher PD-L1 expression, immune infiltration, and IFNγ signatures (14), but the therapeutic efficacy of immunotherapy in patients with METex14 NSCLC remains controversial. A recent retrospective study of 110 patients concluded that immunotherapy may be a valuable option for patients with METex14 NSCLC. Multivariate analysis showed a higher overall response rate (ORR) and longer OS in patients with *TP53* co-mutations who received immunotherapy (15). In fact, 42% of METex14 NSCLC cases harbor *TP53* co-mutations (16). However, the role of TP53 in this context is not fully understood, as specific *TP53* mutations have been correlated with poor outcomes in patients treated with immunotherapy (17).

Recent molecular subtyping of METex14 NSCLC (i.e., MET-driven, FGFR-activated, immune-activated, and bypass-activated) has provided new insights into how inter-tumoral molecular heterogeneity may determine immunotherapy response in these patients (18). The PD-L1-overexpressing MET-driven subtype, characterized by high VAF levels of METex14, exhibits an immunosuppressive TME that may reduce the efficacy of immunotherapy. In contrast, the immune-activated subtype, which exhibits spatial co-option of PD-L1+ cancer cells and pro-inflammatory signatures, shows durable responses to immunotherapy and may benefit from the combination of immune checkpoint inhibitors (ICI) and MET-TKIs (18). Our patient with high VAF levels of METex14 benefited from 14 months of monotherapy with the anti-PD-1 ICI pembrolizumab, but unfortunately developed secondary immunoresistance without changing PD-L1 expression status (19).

Silibinin exhibits activity against BMs, primarily by inhibiting the STAT3/TIMP1 signaling pathway, which is highly active in a subset of RA surrounding metastatic lesions. This helps restore suppressed adaptive and innate immune responses within the BMs microenvironment (7–10). Although the STAT3/TIMP1 axis is considered the main mechanism of action, additional molecular pathways may also contribute to its therapeutic effects (20). These include suppression of angiogenesis, migration, and invasion, as well as the induction of G0/G1 cell cycle arrest and apoptosis. Such effects are likely mediated by downregulation of EGFR and inhibition of its downstream effectors, including JAK2/STAT5 and PI3K/Akt pathway (21). Our group has also identified additional mechanisms, such as inhibition of the metastasis-promoting transcription factor ID3 (22) and interference with the C-terminal domain of HSP90 (23). Additionally, silibinin has been shown to function as an immune checkpoint inhibitor by blocking PD-L1 maturation during post-translational glycosylation in the endoplasmic reticulum, resulting in mannose-rich PD-L1. This altered form of PD-L1 enhances the efficacy of PD-L1 dimer-inducing small molecules and improves the anti-tumor effect of cytokine-activated T-cells (24), demonstrating the immunotherapeutic potential of silibinin. However, further studies are needed to clarify the relative contribution of each pathway. Clinical trials with a controlled arm are also required to account for interpatient variability, especially considering the heterogeneity of MET exon 14 alterations and PD-L1 expression profiles.

Regarding patient perspective, the patient demonstrated a very positive attitude toward the incorporation of silibinin into his treatment plan. He appreciated the convenience of obtaining the product as an over-the-counter supplement, its non-toxic profile, and the noticeable improvement in brain MRI scans and the potential to delay WBRT and its associated side effects, all of which contributed to his overall satisfaction. However, the patient also noted that the treatment is currently inaccessible to economically disadvantaged individuals, as it is not covered by the public health system, making its cost a barrier for some.

## 4 Materials and Methods

This case report was prepared following the CARE Guidelines (Supplementary Table 1).

The initial biopsy was a single CT-guided lung biopsy, which was analyzed by a pathologist. Immunohistochemistry was performed on this sample to assess PD-L1 TPS (Ventana SP263), ALK expression (Ventana D5F3), and ROS1 expression (Ventana SP384). In parallel, a blood sample was collected from the patient for *EGFR* mutation analysis using Cobas *EGFR* Mutation Test v2, due to insufficient tumor tissue for molecular testing.

The jejunal metastasis was surgically resected, and the resected specimen was analyzed by NGS in our molecular laboratory by a molecular biologist. DNA was extracted from paraffin-embedded tissue sections using the cobas^®^ DNA Sample Preparation Kit (Roche Diagnostics). DNA libraries were prepared using the SureSelectXT HS Target Enrichment System (Agilent Technologies), targeting the complete coding region of 43 genes (± 50 base pairs) and intronic regions associated with gene fusions. Then, the prepared libraries were multiplexed and sequenced on an Illumina MiSeq platform. Raw sequencing data were processed with the fastp tool, and reads were aligned to the human reference genome (GRCh37/hg19) using BWA-MEM2. PCR duplicates were removed after alignment. Single nucleotide variants (SNVs) and insertions/deletions (indels) were identified using Mutect2 and Lancet. Copy number variations (CNVs) were detected with CNVKit, and structural variants with Manta. Variants of potential clinical relevance were annotated using databases such as ClinVar, CIViC, chimerDB, and cancer hotspots, and were then reviewed by experienced laboratory experts. The METex14 was subsequently confirmed by the IDYLLA GeneFusions_RUO/1.0 test.

Silibinin supplementation was orally administered using Legasil^®^ (Meda Pharma, Barcelona, Spain), which contains 210 mg of Eurosil 85^®^ (60% of silibinin isoforms) (Euromed, Mollet del Vallés, Barcelona, Spain), a formulation of silibinin with enhanced oral absorption and bioavailability (5). The product is classified as a dietary supplement in Spain and is available without a doctor’s prescription at a price of approximately €20. Titration began with 2 capsules/day (1-0-1) for 3 days; one additional capsule was then added until a maximum dosage of 5 capsules (2-2-1) was reached or toxicity was observed. This dosing regimen was equivalent to 630 mg of silibinin per day.

## 5 Conclusion

This case report provides the first evidence that silibinin may benefit patients with BM by demonstrating durable intracranial clinical activity (> 9 months) of the combination of PD-1-targeted immunotherapy (pembrolizumab) in METex14 NSCLC. Further studies are needed to determine whether the therapeutic effect of silibinin is mediated exclusively through the STAT3/TIMP1 signaling axis or involves additional pathways. Moreover, controlled clinical trials are required to address variability from METex14 alterations and PD-L1 expression. Should clinical trials yield positive results, the integration of silibinin into clinical protocols would be straightforward and cost-effective. Silibinin is affordable, readily available, and convenient to take orally, making it a promising candidate for easy integration into healthcare systems, ensuring both economic feasibility and practical application.

## Data Availability

The original contributions presented in this study are included in this article/Supplementary material, further inquiries can be directed to the corresponding author.
