# Supplementary material for: The STAT3/TIMP1 inhibitor silibinin overcomes secondary immunoresistance to pembrolizumab in brain metastases from METex14 skipping mutated non-small cell lung cancer: a case report
Source: Front Med (Lausanne). 2025 Jul 9;12:1612327. doi: 10.3389/fmed.2025.1612327 (PMC12283722; doi:10.3389/fmed.2025.1612327)
Supplement: Supplementary file 1 [file Supplementary_file_1.docx]

Supplementary Material

**Supplementary Table 1.** CARE Checklist

| **Topic** | **Item** | **Checklist item description** | **Reported** |
| --- | --- | --- | --- |
| Title | 1 | The diagnosis or intervention of primary focus followed by the words “case report” | ✓ |
| Key Words | 2 | 2 to 5 key words that identify diagnoses or interventions in this case report, including "case report" | ✓ |
| Abstract (no references) | 3a | Introduction: What is unique about this case and what does it add to the scientific literature? | ✓ (Abstract) |
|  | 3b | Main symptoms and/or important clinical findings | ✓ (Abstract) |
|  | 3c | The main diagnoses, therapeutic interventions, and outcomes | ✓ (Abstract) |
|  | 3d | Conclusion—What is the main “take-away” lesson(s) from this case? | ✓ (Abstract) |
| Introduction | 4 | One or two paragraphs summarizing why this case is unique (may include references) | ✓ (Introduction) |
| Patient Information | 5a | De-identified patient specific information | ✓ (Case Description) |
|  | 5b | Primary concerns and symptoms of the patient | ✓ (Case Description) |
|  | 5c | Medical, family, and psycho-social history including relevant genetic information | ✓ (Case Description) |
|  | 5d | Relevant past interventions with outcome | ✓ (there is none) |
| Clinical Findings | 6 | Describe significant physical examination (PE) and important clinical findings | ✓ (there is none) |
| Timeline | 7 | Historical and current information from this episode of care organized as a timeline | ✓ (Fig. 1) |
| Diagnostic Assessment | 8a | Diagnostic testing (such as PE, laboratory testing, imaging, surveys) | ✓ (Case Description) |
|  | 8b | Diagnostic challenges (such as access to testing, financial, or cultural) | ✓ (Case Description) |
|  | 8c | Diagnosis (including other diagnoses considered) | ✓ (Case Description) |
|  | 8d | Prognosis (such as staging in oncology) where applicable | ✓ (Case Description) |
| Therapeutic Intervention | 9a | Types of therapeutic intervention (such as pharmacologic, surgical, preventive, self-care) | ✓ (Case Description) |
|  | 9b | Administration of therapeutic intervention (such as dosage, strength, duration) | ✓ (Case Description) |
|  | 9c | Changes in therapeutic intervention (with rationale) | ✓ (Case Description) |
| Follow-up and Outcomes | 10a | Clinician and patient-assessed outcomes (if available) | ✓ (Case Description) |
|  | 10b | Important follow-up diagnostic and other test results | ✓ (Case Description) |
|  | 10c | Intervention adherence and tolerability (How was this assessed?) | ✓ (Case Description) |
|  | 10d | Adverse and unanticipated events | ✓ (Case Description) |
| Discussion | 11a | A scientific discussion of the strengths AND limitations associated with this case report | ✓ (Discussion) |
|  | 11b | Discussion of the relevant medical literature with references | ✓ (Discussion) |
|  | 11c | The scientific rationale for any conclusions (including assessment of possible causes) | ✓ (Discussion) |
|  | 11d | The primary “take-away” lessons of this case report (without references) in a one paragraph conclusion | ✓ (Discussion) |
| Patient Perspective | 12 | The patient should share their perspective in one to two paragraphs on the treatment(s) they received | ✓ (Discussion) |
| Informed Consent | 13 | Did the patient give informed consent? Please provide if requested | ✓ (Informed Consent Statement) |

**CONSENTIMIENTO INFORMADO PARA PUBLICACION DE CASO CLINICO**

He recibido información de mi oncólogo habitual (Dr. J. Bosch-Barrera) del interés de mi caso clínico y que considera que compartir esta experiencia con la comunidad científica puede tener interés para el tratamiento de otros pacientes en una situación similar a la mía.

Por este motivo doy mi consentimiento para que mi caso clínico sea publicado en una revista médica científica.

Comprendo que mi nombre no será incluido en el articulo publicado, y que se intentará con todo esfuerzo mantener mi identidad anónima en el texto y en las imágenes. Sin embargo, comprendo que el anonimato completo no puede ser garantizado, y puede ser posible que alguien quien me conoce me podría identificar del artículo publicado.

Comprendo que el artículo publicado estará disponible en Internet, y que el artículo puede ser reproducido en otros sitios Web o en impreso.

Se me ha ofrecido la oportunidad de leer el artículo.

Firmado:_________________________

Nombre:

Fecha:

**Supplementary Figure 1.** Blank informed consent waived to patient
